# Metabolic Reprogramming and Host Tolerance: A Novel Concept to Understand Sepsis-Associated AKI

**DOI:** 10.3390/jcm10184184

**Published:** 2021-09-16

**Authors:** Juan Toro, Carlos L. Manrique-Caballero, Hernando Gómez

**Affiliations:** 1 Center for Critical Care Nephrology, Department of Critical Care Medicine, University of Pittsburgh School of Medicine, Pittsburgh, PA 15213, USA; jtoro@pitt.edu (J.T.); carlosc@pitt.edu (C.L.M.-C.); 2 Renal-Electrolyte Division, Department of Medicine, University of Pittsburgh, Pittsburgh, PA 15261, USA

**Keywords:** sepsis, metabolism, mitochondria, tolerance, AKI

## Abstract

Acute kidney injury (AKI) is a frequent complication of sepsis that increases mortality and the risk of progression to chronic kidney disease. However, the mechanisms leading to sepsis-associated AKI are still poorly understood. The recognition that sepsis induces organ dysfunction in the absence of overt necrosis or apoptosis has led to the consideration that tubular epithelial cells (TEC) may deploy defense mechanisms to survive the insult. This concept dovetails well with the notion that the defense against infection does not only depend on the capacity of the immune system to limit the microbial load (known as resistance), but also on the capacity of cells and tissues to limit tissue injury (known as tolerance). In this review, we discuss the importance of TEC metabolic reprogramming as a defense strategy during sepsis, and how this cellular response is likely to operate through a tolerance mechanism. We discuss the fundamental role of specific regulatory nodes and of mitochondria in orchestrating this response, and how this opens avenues for the exploration of targeted therapeutic strategies to prevent or treat sepsis-associated AKI.

## 1. Introduction

Sepsis is a common and life-threatening condition defined as a dysregulated immune response to infection associated with severe organ dysfunction [1]. Sepsis is responsible for up to one-fifth of all-cause mortality worldwide, despite ongoing efforts to unveil effective therapeutic targets [2,3]. Mortality during sepsis is directly associated with the development of organ dysfunction [1]. For instance, sepsis-associated acute kidney injury (AKI) occurs in more than 50% of patients with septic shock [4], which increases the risk of death by 30–50% [5,6]. Additionally, close to 30% of survivors never achieve complete renal recovery, rendering them at risk for worsening renal function, chronic kidney disease and shortening their life expectancy [7,8].

Two disruptive notions have changed the pathophysiologic paradigm of organ dysfunction during sepsis. First, Hotchkiss et al. [9] demonstrated that cell death cannot explain the development of organ dysfunction during sepsis because organ dysfunction occurs in the absence of overt necrosis and apoptosis [10]. Second, Langenberg et al. [11] demonstrated that hypoperfusion is not necessary for the development of organ dysfunction, because AKI still occurred in the absence of decreased renal blood flow. These disruptive findings have led to the consideration that cells are successful at defending from inflammatory injury and avoiding death, and to the hypothesis that early organ dysfunction may be the representation of such success. Therefore, understanding the defense mechanisms by which cells and organs defend from injury is a valuable strategy to identify potential therapeutic targets.

## 2. A Framework to Rethink Organ Dysfunction in Sepsis: Resistance and Tolerance

There are two main mechanisms by which hosts defend from infection, resistance and tolerance [12]. The capacity of the immune system to recognize and eliminate or neutralize pathogens, collectively known as resistance [13,14], has been traditionally considered as the cornerstone of defense against infection [15]. Although eliminating the infectious agent is an important survival strategy, limiting tissue injury is paramount to survive sepsis because organ dysfunction is the direct consequence of tissue injury and the primary driver of mortality [1]. Borrowing concepts from evolutionary biology and plant ecology [16], it has now been demonstrated that, like plants, animals and human beings have the capacity to limit tissue injury caused by a given pathogenic load or a given inflammatory response [12]. This capacity is known as tolerance, a key defense mechanism that operates in parallel to, and is characteristically independent of resistance [15].

### 2.1. Resistance

In the context of infection, the capacity of the immune system to limit the microbial burden is known as resistance. During sepsis, pathogen-associated molecular patterns (PAMPs) and damage-associated molecular patterns (DAMPs) are released into the circulation as a result of the interaction with the infectious agent, tissue injury, and the inflammatory response [17]. Both PAMPs and DAMPs, can initiate the immune response by binding to a family of membrane-bound or intracellular receptors known as pattern recognition receptors (PRRs) [13]. When PAMPs and DAMPs bind PRRs, a signaling cascade is initiated, upregulating the genetic expression and release of proinflammatory cytokines, chemokines, reactive oxygen species (ROS), and endothelial activation molecules [13,18]. Although the response elicited by PRRs has been mostly studied in innate and adaptive immune cells, it is now known that non-immune cells such as kidney proximal tubular epithelial cells can express PRRs, predominantly toll-like receptors. Toll-like receptor-4 (TLR-4) is a membrane bound receptor with a high affinity for PAMPs, and for lipopolysaccharide (LPS) present in Gram-negative bacteria [14]. Proximal tubular-epithelial cells express TLR-4 in the basolateral and apical membrane at the level of segment S1 [19]. PAMPs and DAMPs bind to TLR-4 and activate Nuclear factor-kappa B (Nf-κB), which upregulates TEC gene expression of proinflammatory cytokines, such as tumor necrosis factor (TNF-α) and monocyte chemoattractant protein-1 (MCP-1) [14]. These mediators drive the local inflammatory response, inducing injury to kidney TEC through inflammation and oxidative stress [14,20,21]. As TLR-4 are expressed in both apical and basolateral membranes, TEC can recognize DAMPs and PAMPs circulating through peritubular capillaries and filtered through the glomerulus in the tubular fluid, resulting in a ‘double hit’ injury [20,22]. Therefore, protection of the host by the inflammatory response through resistance comes at a cost to the host’s fitness, namely the unintended inflammatory injury inflicted to the host’s tissues, a process known as immunopathology.

During sepsis, the dysregulated immune response to infection makes immunopathology an important contributor to the development of organ dysfunction. The severity of the systemic and local inflammatory response has been associated with organ injury and the development of organ dysfunction. Murugan et al. [23], in a multicenter cohort study, described an association between high levels of circulating proinflammatory cytokines such as interleukin-8 (IL-8) and apoptotic markers such as tumor necrosis factor receptor-1 (TNFR-I) with slower renal recovery and higher mortality in critically ill patients receiving renal replacement therapy (RRT). Additionally, Cho et al. [24] reported an association between higher levels of anti-inflammatory cytokine, such as interleukin-10 (IL-10) and the regulatory T-cell marker sCD25, in the development of sepsis-associated AKI when compared to AKI of other etiologies and to non-septic patients.

### 2.2. Tolerance

Tolerance is a defense mechanism of the host that limits the deleterious effects of infection and immunopathology on host fitness [15]. Tolerance capacity must not be confused with the traditional concept of immune tolerance, which is the ability of the immune system to not recognize and react to self-antigens [13].

**Figure 1:**
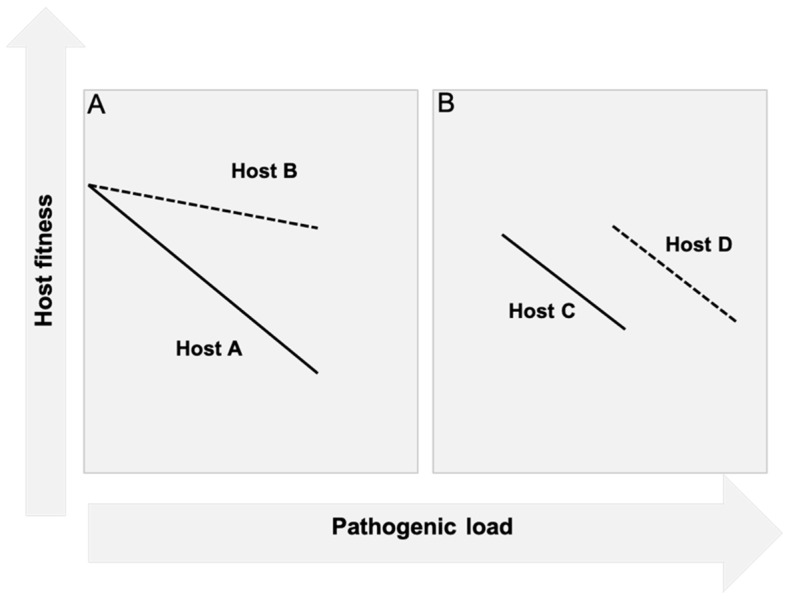
Reaction norm demonstrating the concept of Tolerance and Resistance. (**A**) Host fitness is plotted against bacterial burden for two different hosts (Host A, solid line, and Host B, dashed line). As the figure shows, for the same bacterial burden (same mean pathogen load in x-axis), the health cost (i.e., the change in Host fitness) of such infection is greater for host A than for host B. The slope of the curves is representative therefore, of the Tolerance capacity of each host. (**B**) Host fitness is plotted against bacterial burden once again for two different hosts (Host C, solid line, and Host D, dashed line). Host C and D have the same Tolerance capacity because the slopes of the curves are identical. However, Host D has lower Resistance capacity than Host C because the mean pathogen load is higher.

While the concept of tolerance has been long accepted in plant biology, it was only in 2007 that Råberg et al. [25] demonstrated the existence of this mechanism in animals. Plant biologists studied tolerance by assessing the impact that a specific pathogen load (i.e., colony forming units of bacteria) had on the health status or fitness of the plant (i.e., seed production). They quantified this impact by using plots called reaction norms, which consisted in plotting health status against bacterial load. In reaction norms, the slope of the curve is indicative of the tolerance capacity of the plant to that specific pathogen (Figure 1). Using this approach, Råberg et al. [25] exposed five different strains of mice to different intensities of malaria infection by using different strains of *Plasmodium chabaudi*. They plotted red blood cell density as a surrogate for health status (because a key pathogenic mechanism of malaria is hemolysis) against parasite burden for each strain of mice. The comparison of the slopes of the resulting curves demonstrated differences in the tolerance capacity among strains of mice suggesting differences in tolerance. A clinical correlate in human malaria is the case of patients with the α+-thalassemia mutation, who upon exposure to malaria have less severe anemia and lower mortality than non-carriers despite similar levels of parasitemia, suggesting a higher tolerance capacity [26,27].

Using a murine model of sepsis, Larsen et al. [28] demonstrated the role of free heme in the development of AKI. They also demonstrated that the administration of the heme binder, hemopexin, decreased the development of organ dysfunction and death without altering the bacterial burden, suggesting protection was mediated by a tolerance mechanism. In humans, lower levels of hemopexin were associated with higher mortality, suggesting this mechanism could bare clinical relevance.

Tolerance can take the form of a multitude of mechanisms, most of which have not been discovered. Importantly, the protective effects of any specific tolerance mechanisms seem to be disease- and even pathogen-specific. Using murine models, Wang et al. [29] compared the effect of host anorexia, a stereotypical feature of acute infections, on outcomes after sepsis induced by *Influenza* sp. or by *Listeria monocytogenes*. The authors demonstrated that anorexia was protective during bacterial sepsis, but harmful during viral sepsis. Therefore, the translation of potential tolerance therapeutic targets to the bedside must be performed considering the specificity of any given mechanism to the underlying disease process and avoid the ‘one-size fits all’ approach.

## 3. The Fundamental Role of Cellular Metabolic Reprogramming during Sepsis

Metabolic reprogramming in response to injury is an evolutionarily conserved mechanism of cell survival. The simplest example of the importance of metabolic reprogramming was described by Buck et al. [30] while studying the cellular response to hypoxia. They showed that the exposure of turtle hepatocytes to hypoxia triggered a hierarchical shut down of energy consuming processes, reducing oxygen and energy consumption up to 10-fold, while sparing vital functions such as intracellular ionic balance [30]. Subramanian et al. [31] demonstrated that this hierarchical ‘downregulation’ mechanism is preserved across species, showing that rodent hepatocytes exposed to sublethal, prolonged hypoxia also decreased ATP and oxygen consumption while maintaining the activity of the Na+/K+ ATPase pumps which are critical for intracellular ionic balance.

Cell function and survival are intimately related to the capacity of the metabolic machinery to generate sufficient usable energy and to match energy availability to demand. Two key metabolic pathways exist by which cells transform nutrients into usable energy, namely adenosine triphosphate (ATP): oxidative phosphorylation (OXPHOS) and glycolysis. Compared to glycolysis, OXPHOS is a more efficient metabolic pathway in terms of ATP generation, and therefore it is the default metabolic phenotype of most cells. This is particularly important in cells that demand high levels of energy, like kidney TEC. For instance, proximal TEC have the daunting and energy consuming task of reabsorbing 60–70% of the sodium load contained in the ~170 L/day fluid filtered through the glomerulus [32]. It is no surprise then that proximal TEC have the highest content of mitochondria in the kidney, and that their content of mitochondria is second only to that of cardiac myocytes [33]. Conversely, some cells that lack the machinery to execute OXPHOS (e.g., erythrocytes) or are constantly exposed to low oxygen tension, like TEC in the kidney medulla, usually default to glycolysis as their primary form of energy generation. However, beyond the vital function of maintaining energy balance, metabolic reprogramming between OXPHOS and diverse forms of glycolysis is a resource that cells use to repurpose cell function, withstand injury, and determine the course of tissue repair.

## 4. The Cellular Panic Button: Reprogramming from Oxidative Phosphorylation to Aerobic Glycolysis and Back

**Figure 2:**
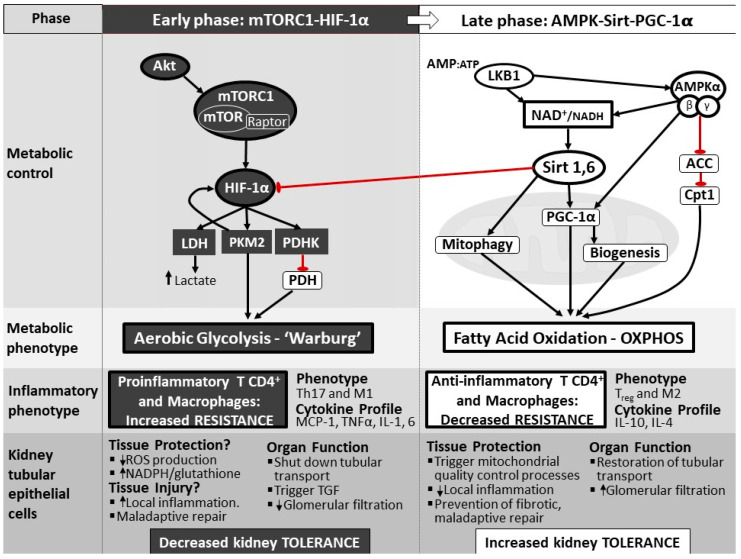
Hypothetical scheme of the regulatory nodes driving metabolic reprogramming toward aerobic glycolysis (**left panel**) or oxidative phosphorylation (**right panel**) in the kidney tubular epithelial cell. During early inflammation, activation of HIF-1α through the Akt/mTORC1 pathway, results in the expression of PKM2 and PDHK, which inhibits the conversion of pyruvate into Acetyl CoA, and therefore its entry into the mitochondria. This early phase is followed by a late phase, where the cell returns to the default metabolic phenotype which relies on OXPHOS for energy production. This switch is led by the cooperative activation of AMPK, PGC-1α, Sirt 1 and Sirt 6. AMPK, PGC-1α and Sirt 1 are the key regulatory nodes that promote fatty acid oxidation and glucose oxidation (i.e., OXPHOS), whereas Sirt 6 may have a role in inhibiting the actions of HIF-1α on glycolytic enzyme expression. ACC, acetyl co-enzyme A carboxylase; AMP, adenosine monophosphate; ATP, adenosine triphosphate; MCP-1, monocyte chemoattractant molecule 1; NAD+, nicotinamide adenine dinucleotide (oxidized); NADH, nicotinamide adenine dinucleotide (reduced); TH17, type 17 T helper; TNF, tumor necrosis factor; Treg, regulatory T cell. Modified from ( [40] and Pool R; Gomez H; Kellum J.A; Mechanisms of Organ Dysfunction in Sepsis. Crit Care Clin. 2018 Jan, 34(1), 63–80).

During the early stages of infection, reprogramming of the metabolism is necessary for monocytes and lymphocytes to change their inflammatory phenotype and mount an appropriate inflammatory response. Native macrophages, dendritic cells, and T-lymphocytes switch metabolism from OXPHOS to glycolysis, regardless of the presence of hypoxia as a more favorable phenotype to promote cellular growth and proliferation [34,35,36,37,38]. This type of glycolysis is known as aerobic glycolysis, and was first described in cancer cells as the Warburg effect [39]. Lymphocytes and monocytes that shift to aerobic glycolysis morph into proinflammatory phenotypes, namely T-helper 17 (Th17) and M1 macrophages, inducing the secretion of proinflammatory cytokines and contributing to the inflammatory response (Figure 2) [35,40]. Cheng et al. [41] have suggested that the switch to aerobic glycolysis in immune cells is necessary to mount an appropriate inflammatory response, and that failure to do so may result in immunoparalysis, high risk of infection, and increased mortality. The authors described that the inhibition of mammalian target of rapamycin (mTOR)-pathway in the early stage of sepsis with metformin decreased the production of proinflammatory cytokines (TNF, IL-1B, IFN-γ) in monocytes exposed to β-glucan. Moreover, when the same model was replicated in mice, higher mortality was observed [41].

Insight into the mechanisms involved in reprogramming metabolism toward aerobic glycolysis comes from studies in cancer and immune cells. In actively proliferating tumor cells, macrophages, and dendritic cells, the switch to aerobic glycolysis is driven by the activation of the mTOR complex 1 (mTORC1) which stabilizes a key transcription factor, the Hypoxia inducible factor 1α (HIF-1α). HIF-1α is the key regulating node that reprograms the cellular machinery to aerobic glycolysis in four steps. First, it enhances glucose uptake by inducing the expression of the glucose transporter 1 (GLUT1) [35]. Second, it creates a bottleneck at the last step of glycolysis by decreasing the conversion of phosphoenolpyruvate to pyruvate, through the expression the enzyme Pyruvate kinase M2 (PKM2) [35,42]. Third, it inhibits the entry of pyruvate into the mitochondria. By increasing the expression of Pyruvate dehydrogenase kinase (PDHK), activation of HIF-1α inhibits pyruvate dehydrogenase (PDH), which is necessary to convert pyruvate to Acetyl CoA [43,44]. Fourth, it promotes the conversion of pyruvate into lactate by enhancing the expression of lactate dehydrogenase A (LDH, Figure 2). This switch has been linked to an adaptive mechanism through which the cell appears to meet the energetic demands required to undergo proliferation and to mount an adequate inflammatory response [37]. Similarly, in LPS induced M1 macrophage activation, an increased expression of the isoform of phosphofructokinase-2 (u-PFK2) promotes Warburg metabolism, resulting in citrate and succinate accumulation [45]. These metabolites induce the production of nitric oxide (NO), ROS, prostaglandins, and IL-1β respectively, which are indispensable mediators for an adequate immune response [46,47].

Data supporting the existence of this metabolic switch towards aerobic glycolysis in TEC in response to sepsis is still limited. In the context of polycystic kidney disease and in atrophic TEC after ischemic injury, aerobic glycolysis is driven primarily by the Akt/mTORC1/HIF-1α pathway [48,49], suggesting a similar mechanism to that described in inflammatory cells. Using gas chromatography/mass spectrometry analysis of kidney biopsies obtained 8 h after the induction of sepsis by cecal-ligation and puncture (CLP), we have shown an increase in glycolytic intermediates and a decrease in intermediates of the tricarboxylic acid cycle (TCA) suggestive of a switch in kidney metabolic phenotype toward glycolysis [50]. Further supporting the presence of this shift during sepsis and inflammation, Tran et al. [51] in an LPS-induced sepsis model, demonstrated that gene expression of OXPHOS mediators is suppressed in early LPS-induced AKI.

Although significantly less efficient than OXPHOS, entering glycolysis may allow TEC to enter a ‘shut-down’ mode, whereby the cell reprioritizes energy consumption to survival functions, at the expense of organ function to survive. The kidney tubule therefore shuts down ion transport, which, by increasing the chloride concentration in tubular fluid, activates the tubuloglomerular feedback, which in turn decreases glomerular filtration and results in AKI [22,52]. Schmidt et al. [53] provided evidence of this mechanism of reprioritization of energy consumption in the kidneys of rodents exposed to diverse inflammatory injury. The authors demonstrated that, in the kidneys of rodents exposed to PAMPs (e.g., LPS) or DAMPs (e.g., IL-6 or TNF), there was a profound downregulation in the expression of membrane-bound energy-requiring ion transporters. This is analogous to the cardiac response to ischemia, where cardiac myocytes shift metabolism to glycolysis and decrease contractile function in order to survive. Clinically, this manifests as myocardial stunning, leading to a low cardiac output state and heart failure. It is important to clarify, however, that aerobic glycolysis is not an absolute requirement for cells to reprioritize energy consumption. Indeed, very well-known promoters of OXPHOS, like AMP-activated protein kinase (AMPK), are capable of shutting down energy consuming processes in the context of cellular energy imbalance. On the other hand, although this clinically characterizes organ dysfunction during sepsis, functional shut-down may be the manifestation of protective mechanisms at play early in the course of the syndrome.

While immune cells benefit from diverting metabolism to aerobic glycolysis, it is less clear that this metabolic switch is advantageous to the TEC during sepsis. Theoretically, the switch to aerobic glycolysis may provide TEC with mechanisms to cope with oxidative damage. Increased ROS production can disrupt energy production in mitochondria and cause injury to the mitochondrial and cell membranes, ultimately leading to the activation of apoptosis by the release of cytochrome C [54]. By limiting OXPHOS, TEC decrease the production of ROS by mitochondria. In addition, the diversion of a substrate towards the pentose phosphate pathway by PKM2 and the activation of the key enzyme glucose-6-phosphate dehydrogenase [55] bolsters antioxidant defense mechanisms by increasing NADPH availability [56], which is necessary to reconstitute reduced glutathione, the primary defense mechanism against mitochondrial ROS (i.e., H_2_O_2_) [57]. In addition, aerobic glycolysis could provide the necessary substrates to sustain cell replication (e.g., fatty acids, nucleotides, amino acids) and enough energy supply to maintain vital processes, such as ionic balance, mitophagy, and mitochondrial biogenesis [39].

However, an important body of evidence suggests that a switch to aerobic glycolysis may be harmful for TEC, the kidney, and the host. This raises the possibility that a switch to aerobic glycolysis in TEC early during sepsis may be a manifestation of disrupted mitochondrial function and OXPHOS, rather than a programmed defense mechanism. Innate immune cells require a shift toward aerobic glycolysis to develop memory to a specific insult and modify the response to future insults (a process known as trained immunity). The development of such trained immunity is protective in rodent models of sepsis [35]. However, the development of trained immunity in kidney TEC may result in hyperactive responses to future stimuli, increasing cell and organ injury. This is particularly important in sepsis, given that sepsis-induced AKI most likely results from diverse, sequential hits, including inflammation, hypoxia, oxidative stress, and nephrotoxins [22]. Zager et al. [58,59,60] demonstrated this by exposing mice to ischemia, nephrotoxins, or ureteral obstruction, and subsequently quantifying the local inflammatory response in the kidney after treatment with LPS or lipoteichoic acid. They found that the kidneys of pre-treated animals responded with higher levels of MCP-1 and TNF than controls. This suggests, that during sepsis, repeated, distinct insults may significantly amplify tubular injury in cells that have switched to aerobic glycolysis and have therefore developed trained immunity.

Preclinical evidence suggests that the direct inhibition of aerobic glycolysis provides a survival advantage to the host in models of rodent sepsis, e.g., treatment with Shikonin, a potent PKM2 inhibitor, 12 h before or 24 h after LPS administration, or 24–48 h after CLP improves host survival [36]. Indirect inhibition of aerobic glycolysis by stimulating mitochondrial OXPHOS has also been shown to be protective during experimental sepsis. Opal et al. [61] and Vacharajani et al. [62] have independently shown that the activation of Sirtuin 1, a key promoter of OXPHOS, increases survival in rodents exposed to experimental sepsis. We have shown that pharmacologic activation of AMPK, a master regulator of energy, promoter of OXPHOS, and inhibitor of Warburg metabolism [63], improves survival in rodents exposed to CLP. More importantly, we have demonstrated that AMPK activation decreases the development of AKI, suggesting that the survival advantage may be associated with reducing organ dysfunction [64].

## 5. The Importance of Shifting Back from Aerobic Glycolysis to OXPHOS

The persistence of aerobic glycolysis impedes monocytes, T cells, and dendritic cells from ‘turning off’ the proinflammatory response, increasing tissue injury through immunopathology. In TEC, there are three main reasons why switching back from glycolysis is necessary. First, similar to inflammatory cells, the persistence of a glycolytic metabolism in kidney TEC is associated with persistent local inflammation and increased injury. Second, restitution of OXPHOS and fatty acid oxidation (FAO) is necessary to restore the expression of ion transporters in the kidney tubule, and therefore to rescue kidney function. Third, TEC that are incapable of restoring OXPHOS and FAO undergo maladaptive repair, resulting in atrophy and fibrosis [49,65], which thus increases the risk of transition from AKI to chronic kidney disease.

In support of this, Kang et al. [66] found that the biopsies of patients and rodents with chronic kidney disease were characterized histologically by atrophy and fibrosis, and molecularly by a profound decline in the expression of OXPHOS and FAO enzymes and an increase in the expression of glycolytic enzymes. Importantly, the authors also demonstrated that this is reversible, because by treating rodents with activators of FAO, e.g., fenofibrate, they were able to limit the development of fibrosis in animals exposed to nephrotoxic injury and abort maladaptive repair programs. Han et al. [67] provided further evidence in support of the impact of the metabolic phenotype on kidney repair after injury. Using a tubule specific LKB1 knock-out rodent system, they found that animals lacking LKB1 which is a key upstream activator of AMPK, had decreased expression of AMPK, peroxisome proliferator activated receptor-coactivator 1α (PGC1α), and FAO-related enzymes, and underwent maladaptive tissue repair, with the subsequent development of fibrosis. Moreover, the use of AMPK and PGC1α agonist in LKB1 null mice mitigated these effects. Therefore, although aerobic glycolysis may have a role in protecting the host during sepsis, particularly due to its effect on immune cells, it is clear that a return to OXPHOS and FAO is necessary to promote adaptive tissue repair and recovery.

## 6. Cellular Molecular Mechanisms to Reprogram Metabolism

During normal physiologic conditions, fatty acid oxidation (FAO), the major form of OXPHOS, is the main source of energy in mature, functional kidney TEC. Therefore, restoring kidney function requires returning to this characteristic physiologic metabolic phenotype. The best evidence detailing the molecular mechanism responsible for the change between aerobic glycolysis and OXPHOS was provided by Liu et al. [68] in human and murine monocytes. The authors demonstrated that the restoration of FAO and OXPHOS required the activation of the peroxisome proliferator activated receptor γ coactivators 1α (PGC-1α) and 1β (PGC-1β). Activation of these promoters increased the expression of the external membrane receptor CD36, a class B scavenger receptor/transporter with high affinity for long chain fatty acids, and carnitine palmitoyl transferase 1 (CPT1), the rate limiting enzyme for mitochondrial long chain fatty acid oxidation. In their study, restoring FAO and OXPHOS also required increased production of NAD^+^ through nicotinamide phosphoryltransferase (Nampt) and the activation of Sirtuin 1 and 6.

In the kidney, Tran et al. [51] demonstrated that, early after LPS stimulation, the expression of PGC-1α declines in parallel to a decrease in kidney function and is only restored ~48 h after. Furthermore, they demonstrated that stimulation of PGC-1α improves the survival of rodents exposed to ischemia reperfusion injury [69] and following LPS [51], suggesting that the stimulation of regulators of FAO and OXPHOS improves outcome and that a shift toward OXPHOS may follow a similar blueprint as in inflammatory cells. Activation of PGC-1α requires a coordinated sequence of phosphorylation and deacetylation by AMPK and Sirtuin 1, respectively [64,67]. AMPK indirectly can also activate Sirtuin 1 by increasing nicotinamide adenine dinucleotide (NAD^+^) availability. Similarly, PGC-1α plays a significant role in the replenishment of mitochondrial NAD^+^ pool via malate-aspartate shuttle (MAS) and NAD^+^ biosynthesis via the salvage pathway. MAS activation by PGC-1 lead the exchange of cytosolic NADH for mitochondrial NAD^+^, maintaining a necessary amount of mitochondrial NAD^+^ to use it as a substrate for TCA cycle and the electron transport chain (ETC), promoting OXPHOS and ATP production [70,71,72]. AMPK inhibits Acetyl CoA Carboxylase (ACC) through an inhibitory phosphorylation. ACC is an enzyme that constitutively inhibits CPT1, and therefore restricts FAO in mitochondria. By inhibiting ACC, AMPK increases CPT1 activity, and thereby promotes FAO. Sirtuin 6, on the other hand, inhibits the effects of HIF-1α, the main driver of aerobic glycolysis, and therefore may be important to restore OXPHOS. Han et al. [40] demonstrated that the deletion of genetic deletion of LKB1 in kidney tubules resulted in progressive tubulointerstitial damage, which was mitigated in animals treated with AMPK and PGC1α agonist. Based on these data, we suggested that the restoration of FAO and OXPHOS in the TEC requires the coordinated interplay of AMPK, Sirtuin 1 and 6, and PGC-1α, as shown in Figure 2.

## 7. The Pivotal Role of Mitochondria in Metabolic Reprogramming during Sepsis

Ion reabsorption in the kidney cortex is an energetically expensive function and therefore, kidney function is intimately related to mitochondrial health. Oxidative phosphorylation takes place in the mitochondria, and therefore the capacity of the TEC to reprogram metabolism hinges on the availability of functional mitochondria. This is important because sepsis is known to cause significant mitochondrial injury, potentially hindering the capacity of the TEC to restore OXPHOS. Moreover, mitochondrial dysfunction is associated with increased risk of organ dysfunction [73].

During sepsis, mitochondrial injury in TEC occurs secondary to oxidative damage [74,75], TLR-4 mediated inflammation [76], and inhibition of the ETC [77] and is characterized by a decrease in mitochondrial mass, mitochondrial fragmentation, disruption of the cristae, and variable degrees of mitochondrial swelling [78]. Furthermore, sepsis alters important mitochondrial quality control functions, which are otherwise critical to maintain a healthy, functional pool of mitochondria. Because of the significant damage that injured mitochondria can cause to the cell, coordinated quality control processes, such as mitochondrial fission, fusion, and mitophagy, provide the cell with the ability to identify, tag, and dispose of such dysfunctional mitochondria. Dysfunctional mitochondria can, for example, split into two daughter mitochondria by fission, and then fuse with healthy mitochondria in an attempt to rescue the dysfunctional pair [79,80]. During sepsis, however, mitochondrial fusion and fission are impaired [81]. In a rodent model of sepsis, Liu et al. [81] described an imbalance in the processes fusion/fission toward increased fission, resulting in increased mitochondrial fragmentation and accumulation of damaged mitochondria.

Mitophagy, a highly specialized form of autophagy by which the cell can identify, target, digest, and remove dysfunctional mitochondria, plays a key role in sepsis-associated AKI [78]. Hsiao et al. [82] found that mitophagy is activated early in the course of sepsis followed by a decline over time. Similarly, Liu et al. [81] quantified the cytochrome c oxidase IV and Microtubule-associated protein 1A/1B-light chain 3 (LC3) as surrogates of autophagy activity in a murine model of CLP. They found a rise in both markers as early as 4 h with a subsequent decrease after 18 h, suggesting an early activation of autophagy during sepsis. Moreover, the stimulation of autophagy with mTOR inhibitors, e.g., anthracyclines or temsirolimus, decreases AKI and mortality, whereas the inhibition of autophagy is associated with worsening apoptosis in the liver in experimental models of sepsis [83,84,85]. Importantly, the activation of mitophagy has also proven to decrease ROS production along with providing an improvement in mitochondrial respiration, contributing to energy balance [86]. While mitophagy can be protective both as an intrinsic mechanism or a therapeutic target, it is important to underscore that increased mitophagy can also lead to apoptosis.

Although mitophagy results in protection by eliminating dysfunctional mitochondria, it also results in a decrease in mitochondrial density, which may impair the ability of the cell to revert to OXPHOS and FAO from aerobic glycolysis. However, mitophagy is associated with biogenesis, a mechanism by which the cell synthetizes functional mitochondria de novo, which is regulated primarily by PGC-1α. Biogenesis is critical to TEC repair and recovery because it is through this process that the mitochondrial pool is replenished. The stimulation of biogenesis through different pathways results in the protection of renal function and increased survival in murine models of sepsis [87,88], suggesting that biogenesis is implicated in the recovery process of TEC after injury.

## 8. Metabolic Reprogramming: A Therapeutic Target Invoking Tolerance

Identifying tolerance mechanisms as therapeutic targets is an appealing alternative to standard practices in the treatment of sepsis. However, three important points must be considered. First, tolerance and resistance are independent, complementary protective mechanisms cells, organs, and hosts deploy to defend from infection, inflammation, and injury. However, the same underlying mechanism (i.e., a switch to aerobic glycolysis) may invoke tolerance or resistance depending on the cell type where the mechanism is operational and may result in very different effects in terms of cell and host protection. For instance, immune cells require a switch toward aerobic glycolysis to mount adequate immune responses against infection (i.e., an adequate resistance capacity). However, the overwhelming majority of the evidence suggests that switching toward aerobic glycolysis in kidney tubular epithelial cells is harmful and results in worse outcome. Second, timing is important when considering these mechanisms. While enhancement of resistance by switching to aerobic glycolysis is important early during sepsis to provide the host with the capacity to contain and eliminate the invading pathogen, the perpetuation of this phenotype will result in persistent inflammation and increase mortality. By the same token, bolstering tolerance through any specific mechanism may be beneficial early during sepsis, but harmful or innocuous when executed late in the course. Third, tolerance mechanisms are disease-specific and may be even organ-specific. For instance, anorexia can improve or hinder the host’s tolerance capacity depending on whether the host is defending against bacterial vs. viral sepsis, respectively.

With the above in mind, we have recently shown that pharmacologic activation of AMPK, using 5-aminoimidazole-4-carboxamide ribunocleotide (AICAR) or metformin, protects against the development of AKI and increases survival in rodent models of CLP-induced sepsis [64,89]. However, it remains unclear how AMPK activation provides protective effects. We have shown that systemic AMPK activation decreased the expression of kidney endothelial activation markers like the intercellular adhesion molecule-1 (ICAM-1) and decreased kidney microvascular leak and leukocyte adhesion in CLP-induced sepsis, suggesting that one potential protective mechanism may be decreasing microvascular dysfunction [89]. However, it is possible that AMPK activation protects the TEC by promoting mitochondrial health through the activation of the key regulators (i.e., Sirtuin 1, PGC1α) involved in mitochondrial biogenesis and autophagy [64,67]. Although not formally tested, it is possible that protection through AMPK activation may be mediated at least in part by the activation of mitophagy. AMPK inhibits mTORC1 by at least two mechanisms, first by inhibiting Raptor, which is part of the mTORC1 complex, and second, by directly phosphorylating the autophagy inductor Unc-51–like kinase 1 (ULK1) [90,91]. Importantly, we have shown that AMPK activation protects from sepsis-induced AKI by protecting TEC mitochondria. In addition, we showed that AMPK activation preserved kidney metabolic fitness, or the capacity to recruit OXPHOS when metabolically challenged, suggesting that protection may also be conferred through promoting OXPHOS.

AMPK has also been linked to the NAD^+^-dependent mitochondrial deacetylase Sirtuin 3. Sirtuin 3 has been proven to protect from AKI by improving the mitochondrial fusion and respiration [92]. Tan et al. [93] revealed that the aerobic glycolysis inhibitor 2-DG increased autophagy via the Sirtuin 3/AMPK pathway in CLP-induced sepsis mice resulting in protection against sepsis-associated AKI. Morigi et al. [94] suggested a similar protection with activation of Sirtuin 3 in a model of nephrotoxic AKI. Protection from AMPK activation in our CLP model was associated with an increased expression in Sirtuin 3 [64], supporting the findings by Tan et al. [93] and Morigi et al. [94]. These data are consistent to demonstrate that, regardless of the specific molecular mechanism, activation of AMPK during sepsis protects against AKI and decreases mortality.

**Figure 3:**
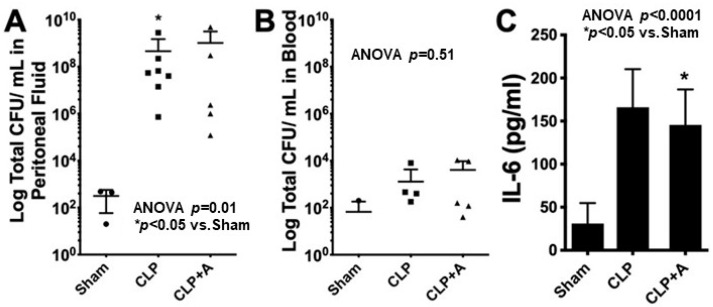
C57/BL6 mice were exposed to either sham surgery (Sham) or cecal ligation and puncture (CLP), and were treated with the AMPK activator 5-aminoimidazole-4-carboxamide-1-β-D-ribofuranoside (AICAR), (CLP+A) or vehicle (CLP). At 24 h, the animals were sacrificed and samples were obtained to assess outcomes. For more details on the experimental design please see the Supplementary Material. There was no difference in the bacterial colony forming units (CFU) between mice treated with AICAR or vehicle in the peritoneal fluid (**A**) or in blood (**B**). In addition, there were no differences in plasma levels of interleukin-6 (IL-6) (**C**). These results suggest that protection through AMPK activation is independent of resistance because there was no effect on bacterial burden, and therefore, must be operating through a Tolerance mechanism. * *p* value < 0.05 when compared to sham.

However, it is unclear if this protection is mediated through a tolerance mechanism. If AMPK activation can limit TEC and kidney injury independently of any effect on resistance, then metabolic reprogramming can be considered a tolerance mechanism. To test this, we subjected mice to CLP and compared the peritoneal bacterial burden of those treated and not treated with the AMPK activator AICAR. In this case, the bacterial burden is a gross representation of the efficiency of the immune system to eliminate bacteria or in other words, a surrogate for resistance. We found that animals treated with AICAR had less AKI and lower mortality than those not treated with AICAR. However, we found no difference in the peritoneal bacterial burden between groups, strongly suggesting that protection through AMPK activation operates through a tolerance mechanism (Figure 3). These data also highlight that the reprogramming of TEC metabolism away from aerobic glycolysis and toward OXPHOS through the activation of AMPK bolsters the tolerance capacity of animals exposed to experimental sepsis, protecting them from developing AKI and from death.

## 9. Conclusions

The recognition that cell death cannot explain the profound organ dysfunction seen in sepsis has led to the consideration that, far from neutral bystanders, cells actively mount a defense that prioritize survival over function. Metabolic reprogramming is a resource that cells use to mount such defense mechanisms, with far reaching implications in terms of cell and organ protection, tissue repair, and restoration of function. The introduction of the concept of tolerance, has well complemented this novel conceptualization of how sepsis induces organ dysfunction. The recognition of metabolic reprogramming as an intrinsic mechanism of self-defense that can reduce the susceptibility to injury through tolerance and maximize the possibility of return of organ function during sepsis has opened a novel field of possible therapeutic approaches to sepsis.

## Data Availability

Data is available at the University of Pittsburgh.

## Supplementary Materials

The following are available online at https://www.mdpi.com/article/10.3390/jcm10184184/s1, Experimental Methods: Metabolic Reprogramming and Host Tolerance: A Novel Concept to Understand Sepsis Associated Aki.

## Institutional Animal Care and Use Committee Statement

The study was conducted according to the ARRIVE guidelines and approved by the Institutional Animal Care and Use Committee (IACUC) of the University of Pittsburgh (protocol number 19105829, approved 10 January 2019).
