# Supplementary material for: Metabolic Reprogramming and Host Tolerance: A Novel Concept to Understand Sepsis-Associated AKI
Source: J Clin Med. 2021 Sep 16;10(18):4184. doi: 10.3390/jcm10184184 (PMC8471000; doi:10.3390/jcm10184184)
Supplement: Supplementary file 1 [file jcm-10-04184-s001.zip › jcm-1313203-supplementary.pdf]

Journal of
Clinical Medicine

MDPI

Supplementary material

Experimental Methods

Metabolic Reprogramming and Host Tolerance: A Novel Concept to Understand Sepsis Associated Aki

Juan Toro [1], Carlos L. Manrique-Caballero [1,2] and Hernando Gomez [1]

[1] Center for Critical Care Nephrology, Department of Critical Care Medicine, University of Pittsburgh School of Medicine, Pittsburgh, PA 15213, USA
[2] Renal-Electrolyte Division, Department of Medicine, University of Pittsburgh, Pittsburgh, PA 15261, USA

Animal experimentation

All experiments were performed in accordance with the National Institutes of Health Guidelines, the ARRIVE guidelines and under protocols approved by the Institutional Animal Care and Use Committee of the University of Pittsburgh (IACUC protocol No. 16119369). All experiments were performed under isofluorane anesthesia, buprenorphine was used to prevent and alleviate pain, and all efforts were made to minimize suffering of the animals.

Ten- to twelve-week-old, male C57BL/6 wild type mice (Charles River Laboratories) were used for the experiment. Animals were randomized to one of three groups: sham (n=3), CLP (n=7), CLP+AICAR (n=5). Animals in the AICAR group were treated with Aminoimidazole-4-carboxamide 1-β-D-ribofuranoside (AICAR, 500mg/kg dissolved in distilled H2O, intraperitoneal (IP), Biovision, Milpitas, CA, USA, #1687) 24h before CLP. Data is displayed as the mean and standard error from the mean (SEM) of the results of each assay of a group of animals sacrificed at each time point. We selected the dose of AICAR based on our previously published data (Escobar, D. A., Botero-Quintero, A. M., Kautza, B. C., Luciano, J., Loughran, P., Darwiche, S., Rosengart, M. R., Zuckerbraun, B. S., and Gomez, H. (2014) Adenosine monophosphate-activated protein kinase activation protects against sepsis-induced organ injury and inflammation. J. Surg. Res. 194, 262–272), and on pilot studies demonstrating greater activation of AMPK with 500 mg/kg vs. 100 mg/kg (data not shown).

Cecal Ligation and Puncture (CLP)

Mice were subjected to CLP as described previously in a dedicated surgical suite in our laboratory.(Escobar, D. A., Botero-Quintero, A. M., Kautza, B. C., Luciano, J., Loughran, P., Darwiche, S., Rosengart, M. R., Zuckerbraun, B. S., and Gomez, H. (2014) Adenosine monophosphate-activated protein kinase activation protects against sepsis-induced organ injury and inflammation. J. Surg. Res. 194, 262–272) Briefly, a 1 cm laparotomy was performed and the cecum was identified and externalized. The cecum was measured from the ileo-cecal valve to the tip, ligated at the ~ 50% mark with a 4-0 sill suture (Surgical Specialties, Mockena, IL, USA, #781B). The cecum was then punctured once (through-and-through) with a sterile 21-gauge needle on the antimesenteric border, gentle pressure was applied to extrude a small amount of feces (~1mm) and then the cecum was returned into the abdomen. The abdominal wall was closed by planes using 4-0 silk (Surgical Specialties, 781B). Sham surgery control animals underwent the same laparotomy, identification and externalization of the cecum, but no ligation or perforation. All surgical procedures were conducted under isofluorane anesthesia (~2%), with no differences in time of exposure between sham and CLP (data not shown) and during the same time of the day (morning). Animals were resuscitated in their immediate post-operative period

with 1 mL of lactate ringers administered subcutaneously, recovered on warming blankets and had free access to food and water. Animals were treated with buprenorphine immediately after recovery from anesthesia, and every 12 hours thereafter for 24h. Intraperitoneal ampicillin sulbactam was administered at 18 hours after CLP or Sham surgery. Animals were sacrificed after performing peritoneal lavage fluid (see below), by increasing inhaled anesthetic (isofluorane) followed by either cardiac puncture for blood collection or cervical dislocation.

Measurement of renal function and IL-6

Plasma Creatinine (Sigma, MAK080), Cystatin C (R&D Systems, MSCTC0), NGAL (R&D systems, MLCN20), and IL-6 (R&D Systems, M6000B) were measured using commercially available kits.

Peritoneal Lavage Fluid and Blood Collection and Bacterial Culture

Peritoneal lavage fluid (PLF) and blood collection. Under anesthesia and aseptic technique, the laparotomy incision was opened approximately 1cm to inject 1.0ml phosphate buffered saline (PBS) into abdominal cavity, followed by gentle massage of the abdomen. Using 1ml syringe with no needle, as much peritoneal lavage fluid (PLF) from the abdominal cavity was collected. Next, the incision was extended to open rib cage and expose the heart. Blood was collected via cardiac puncture with 1ml syringe and 27g needle that have been coated with heparin.

Bacterial culture. Serial dilutions of PLF and whole blood was done using PBS to optimize counting of the number of bacterial colonies. Fifty microliters (50uL) of PLF or whole blood at room temperature were dropped in the center of 5% sheep blood agar plates (Teknova Inc, Hollister, CA, #B0142). Using a sterile rod, PFL and blood were spread evenly over the plate. Plates were then incubated in 37 degree non-CO2 incubator for 24 hours.
